# Tramadol for the treatment of catheter-related bladder discomfort: a randomized controlled trial

**DOI:** 10.1186/s12871-018-0659-5

**Published:** 2018-12-20

**Authors:** Shuying Li, Liping Song, Yushan Ma, Xuemei Lin

**Affiliations:** Department of Anesthesiology, Key Laboratory of Birth Defects and Related Diseases of Women and Children, West China Second Hospital of Sichuan University, Chengdu, China

**Keywords:** Catheter-related bladder discomfort, VAS, Tramadol, Treatment

## Abstract

**Background:**

Intra-operative urinary catheterization frequently causes postoperative catheter related bladder discomfort (CRBD) during recovery. We conducted this study to evaluate the efficacy of tramadol, which with muscarinic receptor antagonist property, as a treatment for CRBD.

**Methods:**

Ninety patients who underwent elective gynecological surgery and complained of CRBD in the (PACU) were randomized into three groups of 30 each. Group A received normal saline, group B 1 mg/kg tramadol, and group C 1.5 mg/kg tramadol. The medication was administered from the Murphy’s dropper with a slow drip, and the severity of CRBD (none, mild, moderate, and severe) and postoperative pain were assessed after 0, 0.5, 1, 2 and 6 h.

**Results:**

The severity of CRBD was reduced in group C compared with that in groups A and B at 1 h, and in groups C and B compared with that in group A at 2 h. The incidence of CRBD was reduced in group C compared with that in groups A and B at 2 h, and in group C compared with that in group A at 6 h. The visual analog scale (VAS) was reduced in group C compared with that in groups A and B at all time intervals. No differences in adverse effects were observed.

**Conclusions:**

Tramadol 1.5 mg/kg was more effective than tramadol 1 mg/kg in treating CRBD and reducing postoperative pain, without significant side effects.

**Trial registration:**

ChiCTR1800016390. Registered on 30 May 2018.

## Background

Urinary catheterization is universally used during surgery, though it can cause discomfort postoperatively. The clinical manifestation of catheter-related bladder discomfort (CRBD) may be discomfort in the supra-pubic region, or be similar to an overactive bladder, which manifests as urinary urgency and urinary frequency with or without urge incontinence [1]. CRBD is so distressing that it can increase postoperative agitation and pain, reduce the satisfaction of hospital stay, extend hospital discharge time, and increase the workload of medical staff [2]. As the postoperative incidence of CRBD can be as high as 47–90%, it often requires clinical intervention [2, 3].

Involuntary contraction of the bladder mediated by muscarinic receptors is the main cause of CRBD. A variety of antimuscarinic agents, such as tolterodine, oxybutynin, gabapentin, pregablin, butylscopolamine, paracetamol, ketamine, and dexmedetomidine, have been used to prevent CRBD, with varying degrees of success [4–11]. Nonetheless, even with medication, the incidence of CRBD is still as high as 32–69%; thus urgent treatment is necessary [6, 9, 12, 13].

There are very few studies on the treatment of CRBD. The therapeutic drugs studied thus far include ketamine, butylscopolamine and hyoscine [14–17]. Tramadol is a centrally acting opioid analgesic that has an antimuscarinic effect. Tramadol pretreatment has been proven to be effective for decreasing the incidence and severity of CRBD [13]. Accordingly, the present study was designed to investigate the efficacy of different dosages of tramadol as urgent treatment for postoperative CRBD and hypotheses that tramadol 1.5 mg/kg was more effective than tramadol 1 mg/kg.

## Methods

This prospective, randomized, controlled, double-blind study was approved by the China Ethics Committee of Registering Clinical Trials and registered in the Chinese Clinical Trial Registry. Written informed consent was abtained from all patients.

Ninety patients with ASA physical status I–II, who underwent elective gynecological operation with 16 French Foley’s catheters inserted and spontaneously complained of CRBD in the PACU were enrolled. Ages ranged from 18 to 60 years. Exclusion criteria included overactive bladder, bladder outflow obstruction, neurogenic bladder, chronic analgesic abuse, morbid obesity, severe hepatic or renal disease, and inability to communicate.

Patients who complained of CRBD (moderate or severe) were randomized into three groups of 30 each using a computer-generated randomized number. Group A received normal saline, group B received 1 mg/kg tramadol, and group C received 1.5 mg/kg tramadol. The medication was administered from Murphy’s dropper with a slow drip by a PACU nurse who was not involved in the subsequent observation. All patients were monitored by ECG, non-invasive blood pressure, and pulse oximetry. The severity of CRBD, postoperative pain, and adverse effects (nausea, vomiting, dizziness, headache, drowsiness) were then assessed at 0, 0.5, 1, 2 and 6 h by an anesthesiologist who was blinded to the treatment.

The severity of CRBD was recorded as follows: none, did not complain of any CRBD even upon asking; mild, revealed only upon questioning; moderate, reported without questioning but was not accompanied by any behavioral response; and severe, stated on their own and followed by behavioral responses such as a strong verbal response, flailing limbs, and even trying to pull out the urinary catheter [4–9]. Postoperative pain was assessed by the visual analog scale (VAS) score, which varied from 0 to 10, where 0 incicated no pain and 10 the worst imaginable pain.

The sample size was estimated based on a previous study with power analysis α = 0.05 and β = 0.1 to detect differences in the proportion of CRBD in a range of 0.1 to 0.6 between three groups; 24 patients were needed in each group [17]. Considering a 20% dropout rate, 30 patients were ultimately included in each group. The severity of CRBD and adverse effects were analyzed by the chi-squared test. Demographic data were assessed by one-way analysis of variance (ANOVA); postoperative pain was considered the repeated variables and was examined by repeated-measures ANOVA. The data were analyzed using SPSS 17.0, and *P* < 0.05 was considered significant.

## Results

A total of 90 patients were recruited in this study, and no patients withdrew. There were no significant differences among the three groups with regard to characteristics of patient, duration of surgery, and intra-operative sufentanil requirement (Table 1).

**Table 1:** Patients characteristics and clinical data

	Group A	Group B	Group C
Number of patients (n)	30	30	30
Age (year)	35.9 ± 7.7	37.0 ± 8.2	36.6 ± 9.0
Weight (kg)	56.7 ± 5.2	55.3 ± 4.4	55.6 ± 5.2
Duration of surgery (min)	118.3 ± 32.8	122.0 ± 34.9	121.2 ± 35.8
Intra-operative sufentanil requirement (ug)	25.5 ± 7.4	26.0 ± 8.0	25.3 ± 8.1

**Fig. 1:**
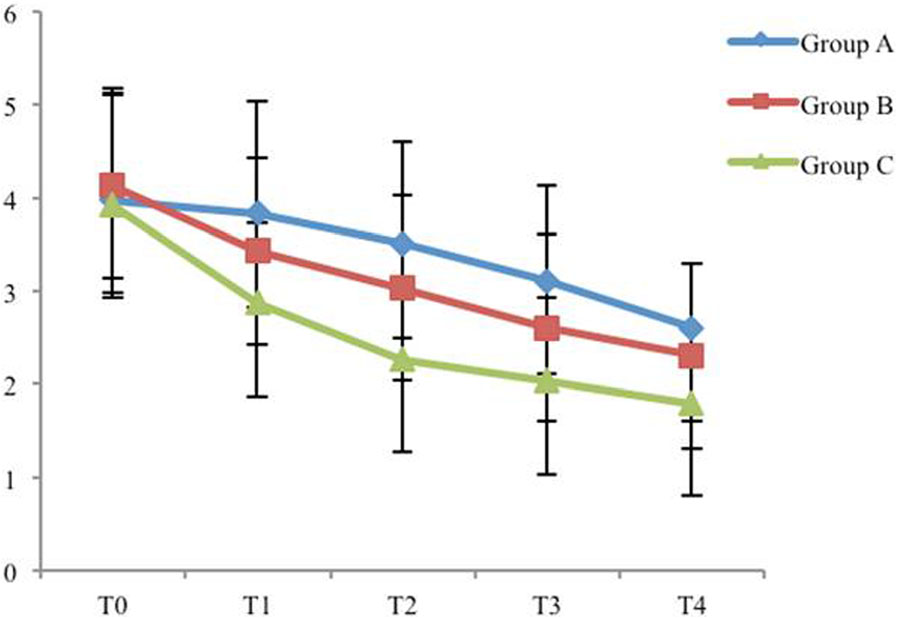
Postoperative pain among the three groups

**Fig. 2:**
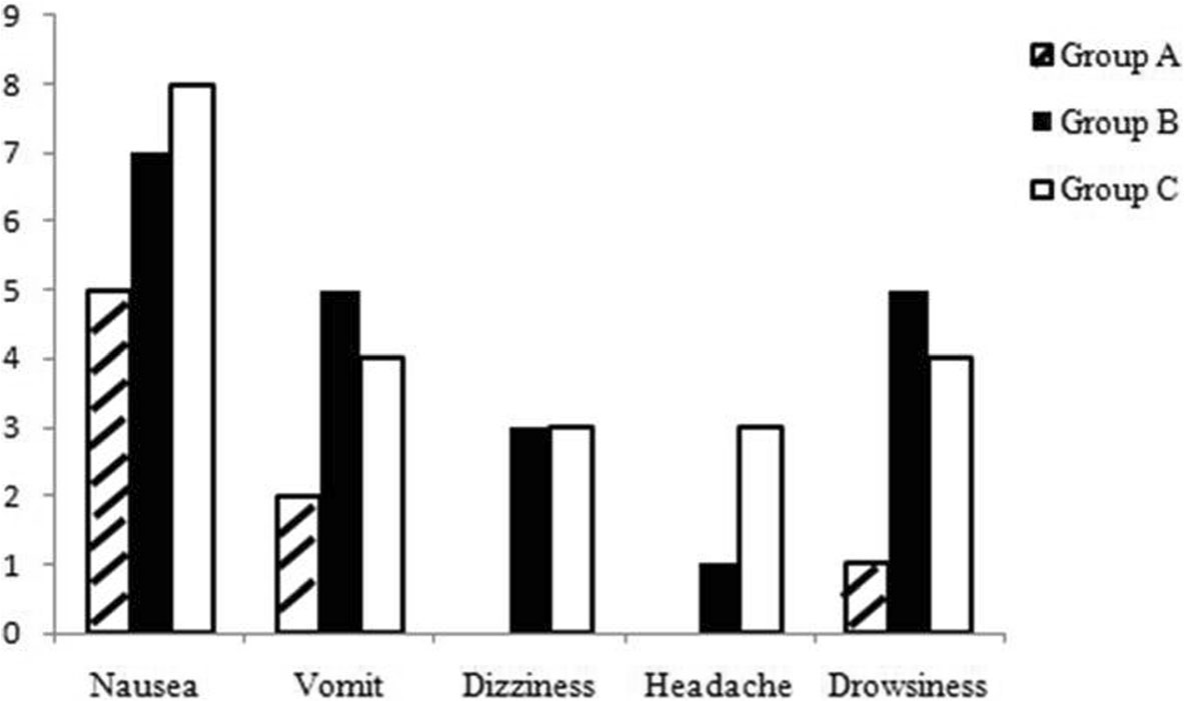
The adverse effects among the three groups

Among the three groups, there was no significant difference in CRBD severity at the first assessment in the PACU and 0.5 h postoperatively. However, the mild severity of CRBD was reduced in group C compared with that in groups A (*P* = 0.002) and B (*P* = 0.046) at 1 h (*P* = 0.008 among the three groups). The incidence of CRBD was lower in group C compared with that in groups A (*P* = 0.024) and B (*P* = 0.01) at 2 h (*P* = 0.012 among the three groups). The severity of CRBD was reduced in groups C (*P* = 0.005) and B (P = 0.008) compared with that in group A at 2 h (P = 0.005 among the three groups). The incidence of CRBD was reduced in group C compared with that in group A (P = 0.005) at 6 h (*P* = 0.013 among the three groups) (Table 2). VAS was reduced in group C compared with that in groups A and B at all time intervals (Fig. 1). There was no difference in adverse effects among the three groups (Fig. 2).

**Table 2:** Incidence and severity of CRBD presented as numbers (n)

Time	T0			T1			T2			T3			T4		
Group	A	B	C	A	B	C	A	B	C	A	B	C	A	B	C
Severity															
No				0	0	0	0	0	7	5	4	13*	16	23	26***
Mild				0	0	6	6	10	14*	8**	18	13	12	7	4
Moderate	26	25	22	26	27	24	22	20	9	17**	8	4	2	0	0
Severe	4	5	8	4	3	0	2	0	0	0	0	0	0	0	0

*P < 0.05 for comparison between group C vs groups A and B. **P < 0.05 for comparison between group A vs groups B and C. ***P < 0.05 for comparison between group A vs group C. T0: 0 h, T1: 0.5 h, T2: 1 h, T3: 2 h, T4: 6 h postoperatively

## Discussion

The severity of CRBD and postoperative pain in patients treated with 1.5 mg/kg tramadol was significantly reduced compared with those given 1 mg/kg tramadol and normal saline. No difference in adverse effects among the three groups was found.

It is clinically common for patients to complain of CRBD when awakening from anesthesia postoperatively and CRBD is identified as a risk factor for postoperative emergence agitation [18]. Severe CRBD usually results in strong vocal response, with shaking of arms and legs; patients will even attempt to pull out the urinary catheter. CRBD can exacerbate postoperative pain and increase the workload of medical personnel. However, CRBD is frequently neglected and left untreated clinically. Management of CRBD is beneficial to improving patients’ comfort and reducing postoperative emergence agitation and pain.

The major two independent predictors of CRBD are male gender and the diameter of the catheter [2]. Operation type is also regarded as one of the risk factors for CRBD, with urological and lower abdominal (such as obstetric and gynecological) surgeries having a higher incidence of CRBD [2, 19]. To eliminate these interference factors, we only enrolled women who underwent elective gynecological operation with a 16F Foley urinary catheter inserted.

Bladder involuntary contraction is mediated by muscarinic receptors, which are stimulated by acetylcholine. The urinary bladder has different types of muscarinic receptors; the M3 receptor leads to direct contraction and the M2 receptor is associated with indirect contraction of the bladder [20, 21]. The major mechanism of CRBD occurs through the M2 and M3 receptors. Tramadol is a centrally acting, synthetic opioid analgesic routinely used to manage postoperative pain. The effects of tramadol include inhibition of noradrenaline (NA), serotonin (5HT) reuptake, and M1 and M3 receptors [22]. Tramadol has been reported to be effictive in preventing the incidence and severity of CRBD [13]. It has also been used as a rescue therapy for moderate or severe CRBD at different dosages, such as 1.5 mg/kg or 50–100 mg [11, 23–25]. The present study evaluated the dose-response effect of tramadol for the management of CRBD. The onset time of tramadol is within 10 min, and the peak effect time is approximately 30 min. Therefore, we observed patients at 0.5 h after the medicine was administered. However, we did not find out any difference among the three groups at o.5 h.

Although adverse effects of nausea and vomiting for tramadol are very common [13, 26], dose–response adverse effects were not observed in this study, and there was no significant difference in the incidence of adverse effects among the three groups. Because the medication was administered from Murphy’s dropper with a slow drip, and the drug concentration was diluted. This study also investigated the dose–response effect of tramadol on postoperative pain relief. The VAS was reduced in group C compared with that in groups A and B at all time intervals; thus 1.5 mg/kg tramadol was more effective than 1 mg/kg tramadol at relieving postoperative pain relief.

There are some limitations in this study. First, we did not evaluate the efficacy of tramadol for all types of surgeries and different surgeries might have different degrees of interference. Moreover, we only observed up to 6 h after the medicine was administred. Although there was no difference in the severity of CRBD among the three groups at 6 h, the half-life of tramadol is 6 h; thus a longer observation time may be necessary. In addition, all the patients enrolled in this study were women, and male gender might have some associated interference.

## Conclusion

In conclusion, tramadol 1.5 mg/kg was more effective than tramadol 1 mg/kg in treating CRBD and reducing postoperative pain without significant side effects.

## Abbreviations

ANOVA: one-way analysis of variance
CRBD: catheter related bladder discomfort
PACU: post-anaesthesia care unit
VAS: visual analog scale
